# Depth profiling of Cr-ITO dual-layer sample with secondary ion mass spectrometry using MeV ions in the low energy region

**DOI:** 10.1038/s41598-022-16042-4

**Published:** 2022-07-08

**Authors:** Marko Barac, Marko Brajković, Zdravko Siketić, Jernej Ekar, Iva Bogdanović Radović, Iva Šrut Rakić, Janez Kovač

**Affiliations:** 1 Ruđer Bošković Institute, Bijenička c. 54, 10000 Zagreb, Croatia; 2 Jožef Stefan International Postgraduate School, Jamova c. 39, SLO-1000 Ljubljana, Slovenia; 3 Jožef Stefan Institute, Jamova c. 39, SLO-1000 Ljubljana, Slovenia; 4 Institute of Physics, Bijenička c. 46, 10000 Zagreb, Croatia

**Keywords:** Mass spectrometry, Mass spectrometry, Experimental nuclear physics

## Abstract

This work explores the possibility of depth profiling of inorganic materials with Megaelectron Volt Secondary Ion Mass Spectrometry using low energy primary ions (LE MeV SIMS), specifically 555 keV Cu^2+^, while etching the surface with 1 keV Ar^+^ ions. This is demonstrated on a dual-layer sample consisting of 50 nm Cr layer deposited on 150 nm In_2_O_5_Sn (ITO) glass. These materials proved to have sufficient secondary ion yield in previous studies using copper ions with energies of several hundred keV. LE MeV SIMS and keV SIMS depth profiles of Cr-ITO dual-layer are compared and corroborated by atomic force microscopy (AFM) and time-of-flight elastic recoil detection analysis (TOF-ERDA). The results show the potential of LE MeV SIMS depth profiling of inorganic multilayer systems in accelerator facilities equipped with MeV SIMS setup and a fairly simple sputtering source.

## Introduction

Secondary Ion Mass Spectrometry using MeV ions (MeV SIMS)^1^ is a fairly new Ion Beam Analysis (IBA) technique that is being increasingly used for the analysis and imaging of organic materials in various fields, such as forensics (fingerprints^2^ and inks^3–5^), cultural heritage (paints^6^), biology (plants^7^ and tissues^8^), etc. Conventional keV SIMS, on the other hand, is a well-established technique used mainly in the analysis and depth profiling of inorganic materials, with the most popular application in the semiconductor industry, i.e. studies of dopant profiles^9,10^, diffusion, corrosion^11^, contamination^12^, etc. It is also convenient for analyzing biomolecules, but with decreased ionization efficiency compared to MeV SIMS^1^ and keV cluster SIMS^13,14^. Techniques developed in order to enhance the ionization efficiency involve the use of high energy gas cluster ion beams (GCIBs) such as C_60_^+^ and Ar_x_ (x = 500–5000)^13,14^ and recently water cluster ions (H_2_O)_n_^+^ (n = 1−10 000)^15,16^, which have proven superior over former.

KeV SIMS is a surface-sensitive technique (ions are detected from a few uppermost monolayers) and can be extended to depth profile analysis by introducing ion sputtering. A variety of applications in SIMS depth profiling arise from high sensitivity to inorganic species and excellent depth resolution^17,18^. Recently, depth profiling of organic films has proven promising by employing either cluster ion beams or low energy Cs^+^ ions for sputtering, due to reduced surface degradation^19^. Depth resolution, a quantitative measure of the depth range, is by convention the sputtered depth measured between 84 and 16% of the maximum yield at an ideally sharp interface between two media^20^. Several different parameters contribute to the profile broadening that originate from instrumental factors, ion beam-sample interactions, and sample characteristics. These include surface roughening caused by sputtering^21^, atomic mixing in the collision cascade^22^, information depth of the technique^23^, non-uniform etching ion beam density in the analyzed area^24^, differential sputtering due to crystalline/amorphous regions^25^, etc. For thicker metallic layers, sputtering-induced roughness is generally the dominating contribution to depth resolution^26^. The best resolution in SIMS (below 5 nm) is achieved with low sputtering ion beam energy^27^ (below 1 keV) and high incidence angle, in combination with sample rotation in order to minimize sputtering-induced roughness^28^. At this point, atomic mixing and information depth are of increasing importance for depth resolution^26^.

On the other hand, modification of the original element distribution due to matrix effects poses a significant limitation in quantitative SIMS depth profiling. This phenomenon arises from the secondary ion yield dependence on the surrounding chemical state (of the matrix). The matrix effect depends largely on experimental conditions, namely the nature of the primary ion, the incident angle, the detected species, and the energy of secondary ions^29^. In data analysis, this is usually tackled with the use of reference materials containing similar matrix^10,30,31^. Many studies have investigated the parameters involved in matrix effects and possible methods for reducing them^32,33^.

MeV SIMS is also surface-sensitive and can in theory be extended to depth profiling. The fundamental difference between keV and MeV SIMS lies in the interaction mechanism of the primary ions with the material. While keV SIMS operates with energies of a few tens of keV through direct energy transfer to the secondary ions via nuclear stopping, MeV SIMS uses heavy energetic ions of a few tens of MeV that interact through electronic stopping with the target’s electronic system. It was shown in our previous work^34^ that lowering the primary ion beam energy from a standardly used few MeV to a few hundred keV enhances the efficiency of secondary ion detection for some inorganic species. Their detection was successful at all three primary ion beam energies used (200 keV Cu^2+^, 440 keV Cu^2+^, and standardly used 5 MeV Si^4+^), exhibiting the expected behavior of secondary ion yield with respect to the primary ion velocity: their yields decreasing with increasing velocity, i.e., decreasing nuclear stopping in the inorganic material, which is a driving force in collisional sputtering. This energy mode was named LE (Low Energy) MeV SIMS. In the present work, LE MeV SIMS depth profiling was explored based on previously confirmed good sensitivity to several inorganic species, in order to compare the achieved depth resolution against keV SIMS, as well as to observe the magnitude of eventual matrix effects, given that specific primary ion beam conditions are introduced in LE MeV SIMS. Up until now, the authors have found no record of an attempt of a dual-beam MeV SIMS depth profiling of inorganic matter in the literature.

This first demonstration of MeV SIMS depth profiling of an inorganic target at low primary ion beam energy opens up possibilities in both fundamental understanding of the impact of the primary ion characteristics on secondary ion yield of inorganic species, as well as expanding the application of MeV SIMS and other types of instruments, such as ion implanters, to perform mass spectrometry of inorganic species within their technical limits. This also presents a novelty for Ion Beam Analysis (IBA) laboratories in terms of the possibility to expand (and possibly improve) the set of existing techniques capable of depth profiling.

## Experimental and methods

A dual-layer Cr-ITO sample was prepared by magnetron sputtering of roughly 50 nm Cr on top of 150 nm ITO (In_2_O_5_Sn), deposited on a soda-lime glass substrate. The sample was first characterized by Time-of-Flight Elastic Recoil Detection Analysis (TOF-ERDA) to determine layers’ atomic content and layer thicknesses. TOF-ERDA measurement was performed by 23 MeV ^127^I^7+^ ions with 20° incidence angle toward the sample surface. TOF-ERDA spectrometer was positioned at an angle of 37.5° toward the beam direction. More details about the used TOF-ERDA spectrometer can be found in the Ref.^35,36^.

The sample was also examined by atomic force microscopy (AFM) in tapping mode at several different places over areas of 2 × 2 μm^2^ to 10 × 10 μm^2^ before and after sputtering, in order to obtain RMS roughness.

KeV TOF SIMS depth profiling was performed on the dual-layer Cr-ITO sample on a TOF.SIMS 5 instrument produced by ION TOF, Germany, at Jožef Stefan Institute in Ljubljana, Slovenia, in a dual-beam mode using 30 keV Bi^+^ analysis ion beam with ion current of 2 pA and 1 keV Ar^+^ etching ion beam with ion current of 127 nA. The analysis was performed over an area of 100 × 100 μm^2^, while etching was done over 400 × 400 μm^2^. The base pressure in the chamber was 5·10^–9^ mbar. Hydrogen was introduced in the analysis chamber at pressure 7·10^–7^ mbar to reduce matrix effects^32^. Mass spectra were obtained in the positive secondary ion mode.

LE MeV SIMS depth profiling was performed on the dual-layer Cr-ITO sample on an in-house MeV TOF SIMS setup described elsewhere^37^. The chosen analysis beam was 555 keV Cu^2+^, having currents in the range of 1–5 fA in pulsed primary ion mode. The analysis covered an area of 300 × 300 μm^2^, under vacuum pressure of 10^–6^–10^–7^ mbar. Sputtering was carried out using PREVAC Ion Source IS 40C1, operating with 1 keV Ar^+^ ion beam and emission current of 10 mA. The ion source was mounted at an angle of 45° with respect to the surface normal. The sputtering beam spot size was approximately 1 × 1 cm^2^, as provided by the manufacturer. During ion sputtering, the sample holder was scanned in two directions in an attempt to homogenize sputtering, thus creating a sputtering area in the shape of a parallelogram with an unspecified area. Ar pressure was ranging from 6·10^–5^ to 3·10^–4^ mbar and the unsuppressed ion current measured on the target was ranging from 6 to 12 μA (changed between cycles). Depth profiling was conducted in a dual-beam mode, in cycles consisting of SIMS analysis of the sample surface by 555 keV Cu^2+^ and sputtering by 1 keV Ar^+^ for 10–20 min. Mass spectra were obtained in a positive secondary ion mode. While generating depth profiles, peak areas of selected secondary ions were normalized to the primary ion current and measurement time.

## Results and discussion

**Figure 1:**
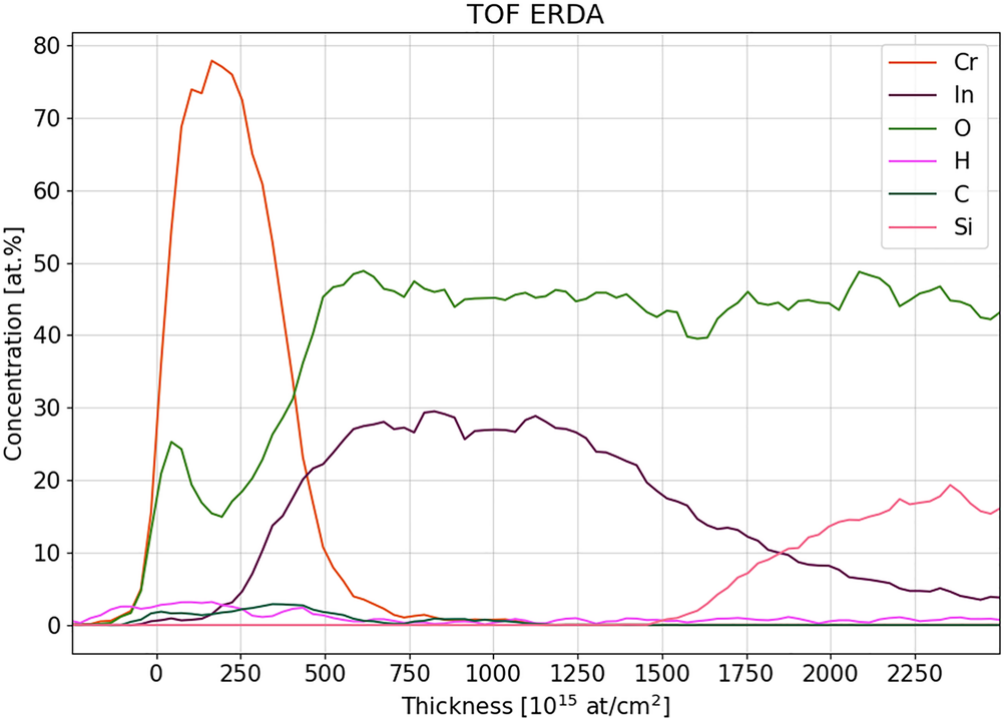
TOF ERDA depth profile of dual-layer Cr-ITO sample on soda-lime glass.

Dual-layer Cr-ITO sample was first evaluated with TOF-ERDA depth profiling. The resulting depth profile of the main species constituting the sample is shown in Fig. 1. Analysis of TOF-ERDA spectra was performed by software Potku^38^. Despite the fact that depth resolution in ERDA deteriorates with depth due to multiple scattering, one can obtain the thickness of each layer by integrating the depth profile, or directly by measuring the distance between half of the maximum of the elemental curve at the layer surface (which marks the beginning of the layer) and at the interface (which marks the end of the layer). Cr and ITO layer thickness is calculated to be 44 ± 3 nm and 154 ± 9 nm, respectively (atomic density of Cr and ITO was considered in conversion from atoms/cm^2^ to nm). It should be noted that because of deteriorating depth resolution (which is roughly 22 nm at the Cr-ITO interface), ERDA has limited capability to resolve eventual structure in the depth profile at the layers’ interface. Since ERDA is a quantitative technique, elemental concentrations are provided in atomic percentages.

**Figure 2:**
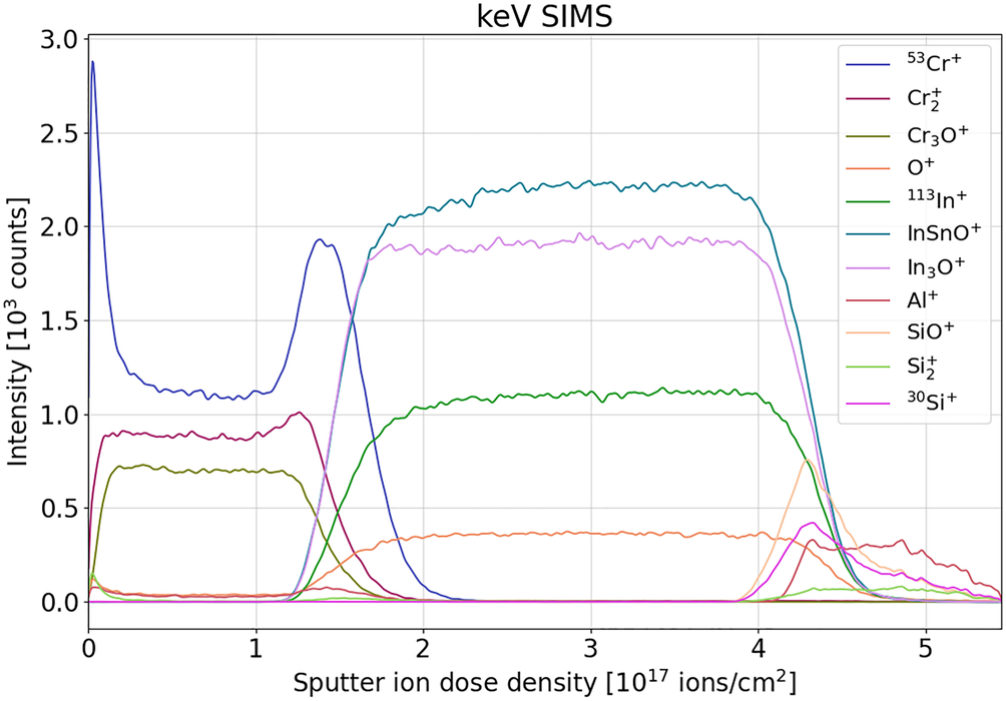
keV SIMS depth profile of selected species from dual-layer Cr-ITO sample on soda-lime glass.

KeV SIMS profiles of selected species with respect to the sputter ion dose density (ions/cm^2^) are shown in Fig. 2. Cr-ITO interface is clearly resolved, and layers’ compositions are mostly uniform. Another ToF SIMS measurement (not shown here) using a 2 keV Cs^+^ etching beam observed the presence of increased CrO^-^ at the surface of the Cr layer and interface with ITO. As a result, partial oxidation of Cr at the Cr-ITO interface causes a matrix effect by enhancing secondary ion yield of Cr^+^, as shown in Fig. 2. Many other oxides were detected as well, but with much lower efficiency.

**Figure 3:**
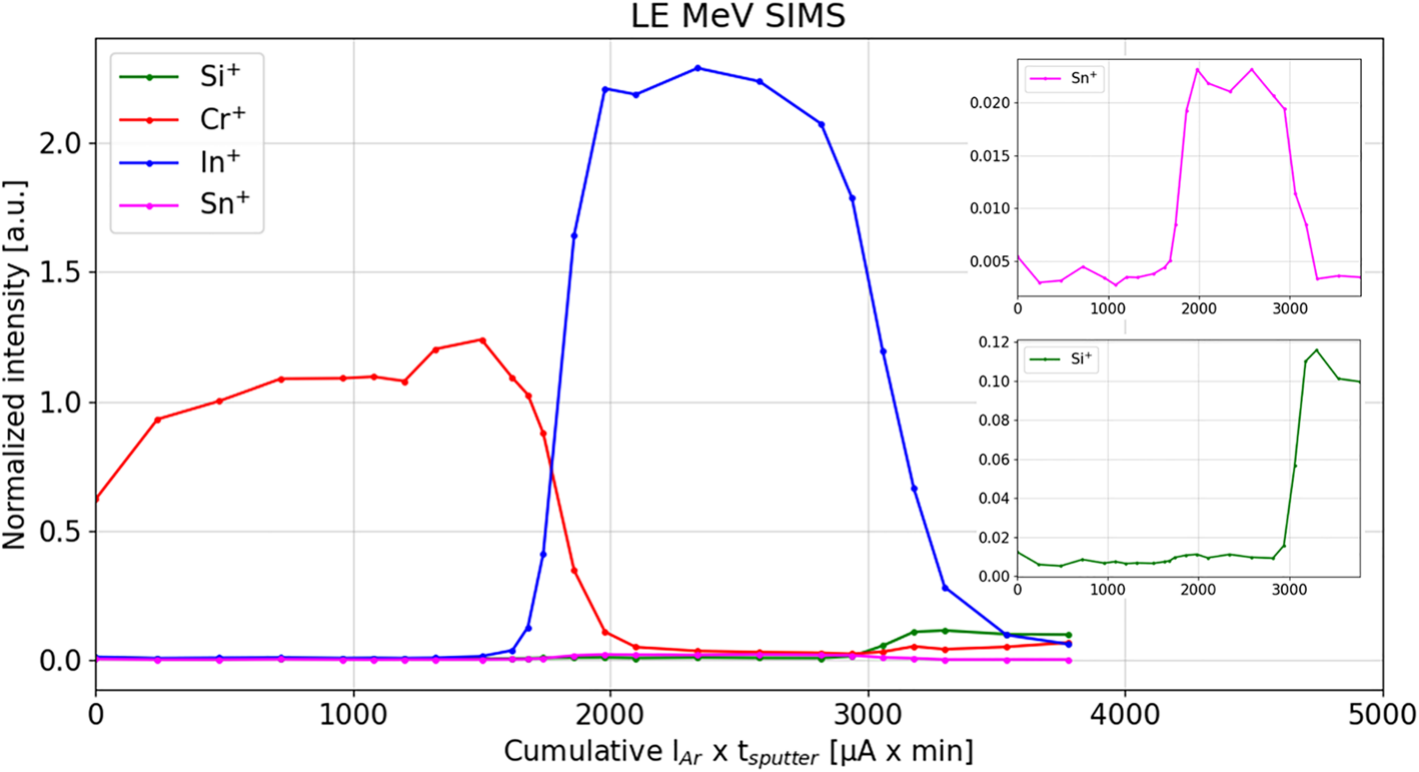
LE MeV SIMS depth profile of detected positive secondary ions from inorganic species from dual-layer Cr-ITO sample on a soda-lime glass. Low intensity ions are presented inside the plot: Sn^+^ (top) and Si^+^ (bottom).

LE MeV SIMS profiles of detected secondary ions from inorganic species are shown in Fig. 3. The x-axis is expressed in “quasi-dose” (cumulative sputtering ion current x sputtering time, per sputter cycle) since the sputter ion dose density could not be calculated due to the inability to precisely define the sputtering area. The main constituents of both layers and substrate (Cr^+^, In^+^, Sn^+^, Si^+^) are detected as positive ions, together with their isotopes with expected abundancies. In the case of Cr and In, the depth profile represents the sum of normalized peak areas of secondary ions of all detected isotopes. The profiles demonstrate significant chemical sensitivity to inorganic secondary ions. No oxides were detected in both positive and negative secondary ion mode, possibly due to lower efficiency to eject oxides with 555 keV Cu^2+^ primary ion beam, and/or due to the presence of a higher amount of background in the mass spectra while operating in the low energy mode. Also, there is generally a significantly lower total secondary ion yield in negative than in positive secondary ion mode. No cluster secondary ions were detected in positive secondary ion mode.

The satisfactory quality of LE MeV SIMS depth profile is reflected in the depth resolution at the Cr-ITO interface that is comparable to that in keV SIMS: the estimated difference in depth between 16 and 84% of the plateau is roughly 10 nm and 11 nm, for keV SIMS (Cr_2_^+^) and LE MeV SIMS (Cr^+^), respectively. This estimation was done by directly converting the x-axes to thickness (in nm) according to ERDA profiles. However, at the end of the dual-layer, the depth resolution in LE MeV SIMS worsens compared to keV SIMS, presumably due to Ar ion gun sputtering conditions during LE MeV SIMS analyses, which could potentially be improved. Another significant observation in LE MeV SIMS depth profile is the reduced matrix effect on Cr^+^ secondary ion yield at the Cr-ITO interface where keV SIMS indicated the presence of oxidized Cr. There is no sharp increase in secondary ion yield at the surface and at the end of the Cr layer as it is observed in the keV SIMS analyses for the Cr^+^ signal, instead the profile is rather steady. However, it is known that monoatomic metallic ions are more prone to matrix effect than cluster ions, hence, in keV SIMS, such layers are usually displayed through metallic dimers or trimers.

AFM of the pre-sputtered sample was performed in several segments. For virgin surface (Cr), an average RMS of 3.6 ± 0.2 nm was obtained. Virgin ITO surface before deposition of Cr layer revealed an average RMS of 3.8 ± 0.1 nm. Because the glass plate came with an already deposited ITO layer, the roughness of the virgin substrate (soda-lime glass) was measured from the substrate backside with an average RMS of 4.6 ± 0.5 nm. In the case of LE MEV SIMS, the crater in the post-sputtered sample revealed an average RMS of 8.1 ± 2.4 nm, whereas for keV SIMS, an average RMS of 9.1 ± 0.4 nm was obtained for the crater. The depth resolution degradation at the end of the ITO layer in LE MeV SIMS compared to keV SIMS is obviously not a consequence of roughness since the crater RMS is comparable to that of keV SIMS. However, it could be attributable to inhomogeneous Ar^+^ sputtering-induced non-horizontal crater bottom since Ar^+^ beam was not focused as in keV SIMS, and the analysis beam covered a fairly large area of 300 × 300 μm^2^.

Another difference between keV and LE MeV SIMS profiles concerns the ratio of the widths of Cr and ITO layer profiles. The width of each profile depends on the sputter yield of that element, given the energy and species of the etching ion beam (atoms/ion). Since profiles from both techniques were generated with the same type of etching beam (1 keV Ar^+^), one would expect a linear dependence between their x-axes. However, the vacuum conditions during sputtering were notably different (7·10^–7^ mbar for keV SIMS, and 6·10^–5^–3·10^–4^ mbar for LE MeV SIMS). There is evidence in the literature that the sputtering yield may be affected by background pressure^39^. Other than that, the authors have found no other explanation for this discrepancy.

## Conclusions

Obtained LE MeV SIMS depth profiles of a dual-layer Cr-ITO sample demonstrate significant chemical sensitivity to inorganic secondary ions, as well as satisfactory depth resolution comparable to that of keV SIMS performed on the same type of sample using the same type of etching beam (1 keV Ar^+^). However, at the end of the dual-layer, depth resolution in LE MeV SIMS worsens compared to keV SIMS, but this is probably due to Ar sputtering conditions, which could potentially be improved by focusing the beam and defining a more uniform beam rastering. A notable revelation was a sign of majorly reduced matrix effect on Cr + secondary ion at the partially oxidized locations in Cr layer (surface and interface with ITO), compared to keV SIMS. This phenomenon is worth further exploring systematically.

One should note that LE MeV SIMS depth profiling is not as straightforward as on the commercial keV SIMS instruments, providing a significantly fewer number of points per sputter cycle, which can be time-consuming. All things considered, all of the observed limitations in LE MeV SIMS profiles seem to be a consequence of the sputtering rather than the analysis conditions. Overall, this work shows the benefit for other IBA laboratories that possess MeV SIMS instrument in expanding its application to inorganic samples by lowering the energy of the primary ion beam, thus gaining multiple orders of magnitude higher efficiencies of inorganic ions and obtaining depth profiles of intermediate-thickness samples with satisfactory depth resolution. Moreover, this can be realized using a relatively low-cost sputtering source.

In theory, this also paves the way for MeV SIMS depth profiling of hybrid organic/inorganic samples such as OLED screens or hybrid solar cells, at least in terms of the ability to detect secondary ions of both organic and inorganic species simultaneously in the low energy mode.
